# Murine RAW Macrophages Are a Suitable Model to Study the CD3 Signaling in Myeloid Cells

**DOI:** 10.3390/cells11101635

**Published:** 2022-05-13

**Authors:** Ranferi Ocaña-Guzmán, Lucero A. Ramón-Luing, Michelle Rodríguez-Alvarado, Timo-Daniel Voss, Tina Fuchs, Leslie Chavez-Galan

**Affiliations:** 1 Laboratory of Integrative Immunology, Instituto Nacional de Enfermedades Respiratorias Ismael Cosío Villegas, Mexico City 14080, Mexico; arocana@iner.gob.mx (R.O.-G.); ramonluing@yahoo.com.mx (L.A.R.-L.); michellerdzalvarado@gmail.com (M.R.-A.); 2 Institute for Clinical Chemistry, University of Heidelberg Medical Faculty Mannheim, D-68167 Mannheim, Germany; timo.voss@medma.uni-heidelberg.de (T.-D.V.); tina.fuchs@medma.uni-heidelberg.de (T.F.)

**Keywords:** CD3 signaling, RAW cells, macrophages, activation, BCG

## Abstract

In recent years, a growing body of evidence has shown the presence of a subpopulation of macrophages that express CD3, especially in the context of mycobacterial infections. Despite these findings, the function of these cells has been poorly understood. Furthermore, the low frequency of CD3+ macrophages in humans limits the study of this subpopulation. This work aimed to evaluate the expression of CD3 in a murine macrophage cell line and its potential for the study of CD3 signaling. The murine macrophage cell line RAW was used to evaluate CD3 expression at the transcriptional and protein levels and the effect of in vitro infection with the *Mycobacterium bovis Bacillus Calmette-Guérin* (BCG) on these. Our data showed that RAW macrophages express CD3, both the ε and ζ chains, and it is further increased at the transcriptional level after BCG infection. Furthermore, our data suggest that CD3 can be found on the cell surface and intracellularly. However, this molecule is internalized constantly, mainly after activation with anti-CD3 stimulus, but interestingly, it is stably maintained at the transcriptional level. Finally, signaling proteins such as NFAT1, c-Jun, and IKK-α are highly expressed in RAW macrophages. They may play a role in the CD3-controlled signaling pathway to deliver inflammatory cytokines such as TNF and IL-6. Our study provides evidence to support that RAW cells are a suitable model to study the function and signaling of the CD3 complex in myeloid cells.

## 1. Introduction

Macrophages are a cell population that comprises a variety of cell subpopulations, each one displaying specific phenotypic and functional characteristics. Macrophages are necessary to maintain an optimal immune response against pathogens. Likewise, they play a crucial role in maintaining tissue homeostasis under sterile conditions [1].

Classically, macrophages are sub-grouped into M1, a more proinflammatory subpopulation, and M2, which are known as anti-inflammatory [2]. However, in 2011, an even smaller subpopulation of monocytes/macrophages was described, which expresses the CD3–T cell receptor (TCR) complex, hereafter called CD3+ macrophages [3]. Although there have been significant advances in the description of this subpopulation, currently, several questions regarding their origin, signaling, and function are still open. It was considered a dogma that the functional expression of the CD3–TCR complex is exclusive to T cells. Structurally, it is composed of eight chains, two of the TCR that could be αβ or γδ, and the CD3 that is constituted by six chains that form two heterodimers CD3Σγ and CD3Σδ, and the CD3ζζ homodimers [4,5]. CD3–TCR complex signaling in T cells is well known. First, the TCR is responsible for identifying the antigen; posteriorly, through phosphorylation of the CD3 chains, several molecules with different functions are recruited, such as protein tyrosine kinases (PTK), including Lck and Fyn (Src family), ZAP-70 (Syk family), linker for the activation of T cells (LAT), and mitogen-associated protein kinase (MAPK), among many others. Subsequently, this signaling activates nuclear factors, including nuclear factor-kappa B (NF-κB), nuclear factor of activated T cells (NFAT), and activator protein 1 (AP-1), to induce the T cell response [6,7,8,9].

It has been described that CD3+ macrophages express TCR that requires the RAG1/RAG2 system to be generated; they were identified for the first time in the granuloma of tuberculosis patients [3]. More recently, evidence with murine models of mycobacterial infections, specifically with the *Mycobacterium bovis Bacillus Calmette–Guérin* (BCG), demonstrated that the transmembrane form of tumor necrosis factor (tmTNF) and its receptor 1 (TNFR1) are indispensable to maintain the presence of this myeloid subpopulation [10,11]. Interestingly, around 15% of human monocyte-derived macrophages (MDMs) are CD3+, and in response to anti-CD3 and anti-TNF stimuli, these CD3+ macrophages exhibit a specific proinflammatory profile [11]. 

Although several reports have demonstrated the relevance of this subpopulation in the context of mycobacterial infections [3,10,11,12,13]; it is important to note that the presence of CD3+ myeloid cells has been described in several other inflammatory pathologies such as atherosclerosis, malaria, and hypersensitivity pneumonitis, among others [14,15,16,17,18], strengthening the hypothesis that this subpopulation is essential to mediate the inflammatory process. 

It is currently unclear whether the CD3 expression on macrophages is constant or cyclic; similarly, it is still unclear which molecules could participate in the CD3 downstream signaling to favor proinflammatory processes. The main issue for the study of this pathway is the low frequency of CD3+ monocytes/macrophages, representing a critical limitation to the design of experimental strategies. Previously, we demonstrated that a fraction of murine RAW 264.7 macrophages (hereafter called RAW) are CD3+ after a BCG infection [10]. This study aimed to clarify if this murine macrophage cell line could be a suitable cell model to study the expression and signaling of the CD3 molecule in myeloid cells.

## 2. Materials and Methods

### 2.1. Raw Cell Culture

RAW cell line was acquired from American Type Culture Collection (ATCC). These cells were unfrozen and were cultured at 1 × 10^6^ cells in a TC-25 flask (Costar, ON, Canada) with RPMI 1640 medium supplemented with 10% FBS (GIBCO BRL, Grand Island, NY, USA), 100 unit/mL penicillin, 10 μg/mL streptomycin and 250 ng/mL amphotericin B (Sigma-Aldrich; Saint Louis, MO, USA).

After a 2-day incubation period (37 °C, 5% CO_2_), cells were collected, and viability was determined by trypan blue exclusion using a TC-20 cell counter system (Bio-Rad, Hercules, CA, USA). The cells in culture were routinely 90 to 95% live cells. The cells were used in passages 10–15, according to previous reports [19].

### 2.2. BCG Preparation and CFU 

M. *bovis* BCG Pasteur strain 1172 P2 (Pasteur Institute, Paris, France) was cultured and grown to the log phase in 7H9 Middlebrook medium. Bacteria were stored at −80 °C in PBS plus 10% glycerol until use. The mycobacterial load was determined by plating serial 10-fold dilutions on agar 7H10 Middlebrook, supplemented with Oleic Albumin Dextrose Catalase (OADC, BD, Franklin Lakes, NJ, USA). BCG colonies were counted after incubation for at least 3 weeks. 

### 2.3. BCG In Vitro Infection

RAW cells (1 × 10^6^) were cultured in 12-well flat-bottomed cell culture plates (Sarsted, Nümbrecht, Germany) and infected with BCG Pasteur strain at MOI 0.1 and 1 for 2 h. At this time, the culture medium was replaced to eliminate non-phagocytosed bacteria. Infected cells were maintained in antibiotic-free RPMI 1640 (10% FBS, 37 °C, 5% CO_2_). Supernatants and cells were collected at 5 and 24 h. Cells were stained for flow cytometry or prepared for protein or nucleic acid analysis, and supernatants were stored at −20 °C until use. 

### 2.4. Flow Cytometry

CD3 and mTNF evaluation were performed by multiparametric flow cytometry in unstimulated RAW cells, stimulated with anti CD3 (24 h) or infected with BCG (5 and 24 h) as described in each section.

For this evaluation, cells were collected and stained for 20 min at 4 °C with mAb to CD3, TNF, CD11b, and F4/80 (Biolegend, San Diego, CA, USA). Fixation procedures were performed to maintain TNF expression, followed by flow cytometric analysis. For intracellular evaluation of CD3, fixation and permeabilization were performed using the citofix/citoperm kit (BD, Franklin Lakes, NJ, USA; Cytofix/Cytoperm™ Plus) according to the manufacturer’s instructions.

Acquisition of cells was performed using a FACS Aria II flow cytometer (Becton Dickinson, Franklin Lakes, NJ, USA, USA) and compensated with a single fluorochrome. Data were analyzed with FlowJo software (Tree Star, San Carlos, CA, USA). The cells used for FMO conditions were stained and acquired in parallel. Dead cells were omitted by the side scatter/forward scatter gating strategy, and isotype-matched control antibodies were used to identify background levels of staining. Typically, 50,000 events were recorded. More details of the antibodies used in this study are shown in Table A1 (Appendix A).

### 2.5. Enrichment of Human T Cells, CD14+ and Obtention of MDM

Blood samples were obtained from donors attending the blood bank at the Instituto Nacional de Enfermedades Respiratorias “Ismael Cosio Villegas”. Peripheral blood mononuclear cells (PBMCs) were isolated from buffy coats using a density gradient as the standard Lymphoprep. In PBMCs, a positive selection for CD14+ cells was performed using magnetic microbeads coated with an anti-CD14 monoclonal antibody (Miltenyi Biotech, Bergisch Gladbach, Germany). The purity of the CD14+ fraction was analyzed by flow cytometry using anti-human CD14, CD2, and CD19 monoclonal antibodies from BioLegend. The efficiency of this process was regularly >90%. To promote the differentiation from monocytes to macrophages (MDMs), 2 × 10^6^ CD14+ cells were cultured in Costar 6-well plates in RPMI-1640 culture medium (GIBCO, Grand Island, NY, USA), supplemented with 2 mM L-glutamine (GIBCO, Grand Island, NY, USA), 100μg/mL streptomycin, 100 IU/mL penicillin, and 10% fetal bovine serum (FBS, GIBCO, Grand Island, NY, USA) for 7 days at 37 °C, 5% CO_2_. Then, MDMs were lysed with 200 µL of lysis buffer (Pierce RIPA buffer; Thermo Scientific, Waltham, MA, USA) to evaluate proteins or suspended in DNA/RNA Shield solution (Zymo Research, Irvine, CA, USA) and stored at −70 °C until use for RNA extraction. More details of the antibodies used in this study are shown in Table A1 (Appendix A).

### 2.6. Splenocytes and Bone Marrow Cells Obtained from C57BL/6 Mice

In this study, we used C57BL/6 wild-type male mice, 8–12 weeks old, housed in animal facility of the Instituto Nacional de Enfermedades Respiratorias “Ismael Cosio Villegas”. All animal experiments were carried out in accordance with institutional guidelines and were approved by the ethical committee on animal experimentation. Bone marrow cells were isolated from the femur of euthanized mice; meanwhile, splenocytes were collected from digested spleen tissue. Bone marrow cells and splenocytes were used as negative and positive controls, respectively, for CD3.

### 2.7. Sorting of CD3+ RAW Cells

To obtain 1.2 × 10^7^ RAW CD3+ cells, we usually sorted 4 × 10^7^ total RAW cells. RAW cells were washed with sterile PBS at 300 g, 10 min, resuspended in 400 µL of staining buffer and labeled with anti-CD3 and anti-F4/80 as previously described. After labeling, cells were resuspended in 5 mL of PBS plus 1% FBS to be acquired and sorted in the FACS-Aria-II cytometer. Instrument configuration was performed by the staff of Flow Cytometry Core Facility of the Instituto Nacional de Enfermedades Respiratorias Ismael Cosío Villegas in Mexico City. The purity of the CD3+ fraction was confirmed by analyzing a sample of 1 × 10^5^ cells by flow cytometry. The efficiency of this process was routinely >90%. 

### 2.8. RAW Cells Stimulated with Anti-CD3 mAb 

Indicated wells were coated with the anti-CD3 antibody or isotype control at 1 µg/mL (Biolegend, San Diego, CA, USA) in sterile PBS and incubated at 5% CO_2_ at 37 °C overnight a day before the experiment. Unbound anti-CD3 was removed on the day of the experiment, then 5 × 10^5^ of total or sorted RAW cells were plated and incubated for 24 h in RPMI-1640 culture medium (GIBCO, Grand Island, NY, USA), supplemented with 2 mM L-glutamine (GIBCO, Grand Island, NY, USA), 100 μg/mL streptomycin, 100 IU/mL penicillin, and 10% fetal bovine serum (FBS, GIBCO, Grand Island, NY, USA) for 24 h at 37 °C, 5% CO_2_. Supernatant and cells were collected for ELISA and WB analysis.

### 2.9. RNA Extraction and q-PCR

Uninfected and infected RAW cells at 0, 5, and 24 h post-infection were suspended in DNA/RNA Shield solution (Zymo Research, Irvine, CA, USA) and stored at −70 °C until use for RNA extraction. Total RNA was obtained with the RNeasy Micro Kit (Qiagen, Hilden, Germany), and genomic DNA contamination was eliminated using RNA-Free DNAse Set (Qiagen, Hilden, Germany) according to the manufacturer’s instructions. Nucleic acid quantification was performed with the Qubit™ assay kit and Qubit 2.0 Fluorometer (Life Technologies, Waltham, USA). RNA in amounts of 500 ng and 100 ng (from RAW cells) or 100 ng (from human MDM and T cells) was used for cDNA conversion using High-Capacity cDNA Reverse Transcription Kit (Applied Biosystems, Waltham, USA) in a reaction volume of 20 µL following the manufacturer’s guidelines. Relative gene expression was evaluated by quantitative real-time PCR (qPCR) using the following TaqMan probes for mouse: TNF (Mm00443258_m1), CD3 (CD3e gene) (Mm01179194_m1); 18S (Rn18s, Mm03928990_g1), and ACTB (β-actin) (Mm00607939_s1). Furthermore, TaqMan probes used for human genes were TNF (Hs00174128_m1), CD3 (CD3e gene) (Hs01062241_m1), 18S (18S ribosomal RNA gene) (Hs03928990_g1), and ACTB (β-actin) (Hs01060665_g1). Single reactions were prepared with the Maxima Probe/ROX qPCR Master Mix (Thermo Fisher Scientific, Waltham, MA, USA), diluting 3 to 1 of cDNA. Amplifications were performed in duplicate under the following thermal conditions: 95 °C for 10 min, followed by 40 cycles of 60 °C for 1 min and 95 °C for 15 s in the Step One Plus Real-Time PCR System (Applied Biosystems, Carlsbad, CA, USA).

Relative gene expression (R.E.) was determined using the ΔΔCT method to calculate the n-fold change for each target gene under each experimental condition. Results were normalized to their corresponding endogenous controls depending on origin cells (murine or human); thus, ACTB and 18S were specific for murine or human. Furthermore, gene expression was calculated relative to the corresponding reference group; for assessment of the anti-CD3 effect at different times, we used unstained RAW cells as the reference group, and for evaluation after BCG infection, uninfected RAW cells, where 2^−ΔΔCT^ = 1. To compare human and mouse CD3 gene expression, first, the ΔCT was calculated considering normalizing to their corresponding housekeeping gene for mouse or human origin as described above. Then, relative expression was determined using uninfected-MDM.

### 2.10. Immunoprecipitation

After removing the medium, RAW cells were washed in PBS and lysed in ice-cold Pierce IP lysis buffer (Thermo Scientific, Waltham, MA, USA) containing Protease Inhibitor Cocktail P8340 (Sigma-Aldrich, Fluka, MO, USA) for 15 min at 4 °C. Lysates were harvested and centrifuged at 13,000× *g*/10 min to eliminate nuclei. Then, IP was performed using Dynabeads Protein G Kit and monoclonal antibodies for mouse CD3 (BioLegend, San Diego, CA, USA). IP was realized following the manufacturer’s guidelines, and precipitated proteins were then analyzed by electrophoresis and posteriorly by immunoblot.

### 2.11. Western Blot (Immunoblot)

Cells were lysed on ice for 10 min using Pierce RIPA buffer (Thermo Scientific, Waltham, MA, USA) containing phosphatases and protease inhibitor (Roche, Basel, Switzerland). Protein quantification was performed in lysates with the Pierce BCA assay protein kit (Thermo Scientific, Waltham, MA, USA), following the manufacturer’s guidelines. Then, lysates were boiled for 10 min in 2× SDS Laemmli sample buffer. Usually, 20–25 micrograms of protein extract were separated by SDS–PAGE, transferred onto nitrocellulose membranes, and analyzed by immunoblotting with mAbs for NFAT, IKK-α, pC-Jun, NF-kB, and pCD3ζchain. β-Actin, β-Tubulin, or GAPDH were used as load control. Western blot analysis was performed using ImageLab software V.6.1. (Bio-Rad, Hercules, CA, USA). Volume boxes were used to define protein bands in samples and controls. Data were normalized using housekeeping proteins as loading controls. This analysis was performed to obtain relative comparisons between different culture conditions. More details on the antibodies used in this study are shown in Table A1 (Appendix A).

### 2.12. Cytokine Measurement in Cell Culture Supernatants

Collected supernatants were thawed and analyzed by enzyme-linked immunosorbent assays (ELISA) to quantify cytokines such as IL-6 (Mouse IL-6 ELISA MAX Standard set Cat. No. 431301) and TNF (Mouse TNF-α ELISA Max Deluxe set Cat. No. 430904). The assays were performed following the manufacturer’s protocols (BioLegend, San Diego, CA, USA). 

### 2.13. Statistical Analysis

Data analysis was performed using GraphPad Prism 9 (GraphPad Software, La Jolla, CA, USA). Data are presented as mean ± standard deviation (SD) and considered non-parametric. Multiple comparisons were performed with Kruskal–Wallis and corrected using Dunn’s test. When data are the means of means, they are presented as mean ± standard error of the mean (SEM), and the multiple comparisons were performed with one-way ANOVA followed by Bonferroni test. *p*-values < 0.05 were considered statistically significant.

## 3. Results

### 3.1. The Macrophage Cell Line RAW Expresses the CD3-Σ Chain

**Figure 1:**
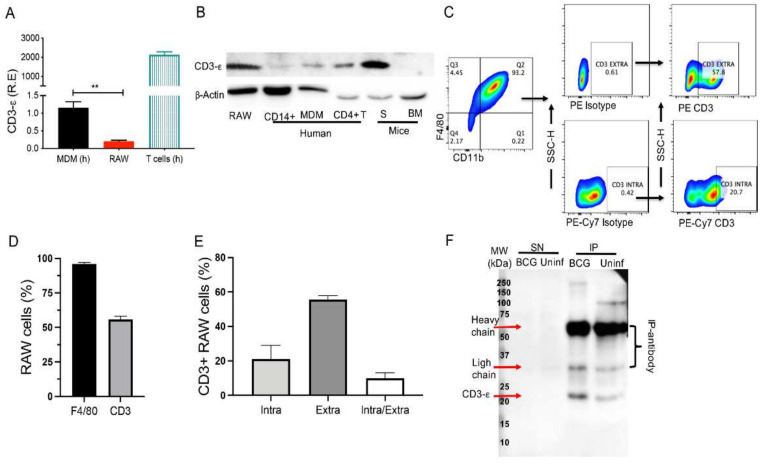
CD3 is expressed on RAW cells. (**A**) RNA of human T cells and monocyte-derived macrophages (MDM) and RAW cells were obtained to assess relative expression (R.E) of CD3-Σ by qPCR. Graphic shows murine and human data; however, each one was normalized to its corresponding endogenous control. (**B**) Immunoblot analysis of CD3-Σ identification in RAW cells, human monocytes (CD14+), MDM, human CD4+ T cells, murine splenocytes (S), and murine bone marrow cells (BM). β-actin was used as a loading control. (**C**) Representative dot plot showing analysis strategy to identify intra- and extracellular CD3 expression in RAW cells by flow cytometry. (**D**) Frequency of RAW cells positive for F4/80 and CD3-Σ obtained by cell surface staining and evaluated by flow cytometry. (**E**) Frequency of RAW cells positive for intracellular or extracellular CD3 or both. (**F**) Representative immunoblot analysis of samples immunoprecipitated with a system of protein G coupled to magnetic beads and monoclonal anti-CD3. *n* = 5 independent experiments (for flow cytometry), *n* = 3 independent experiments by duplicate (for qPCR), (**B**,**F**), a representative image of 3 independent experiments. Bars represent the mean of means ± SEM (to (**A**)) or the mean value ± SD (to (**D**,**E**)). One-way ANOVA followed by the Bonferroni test were used for qPCR analyses. ** *p <* 0.01.

Our first goal was to analyze whether the RAW cells express the CD3 complex. The CD3-Σ chain was evaluated at the transcriptional level in RAW cells, and human T cells were used to reference the maximum expression (Figure 1A). Previously it was shown that human MDM express CD3; thus, we included human MDM. Our results confirmed that RAW cells have a CD3 expression at the transcriptional level, but the relative expression (R.E) of CD3 expression was lower compared to that of MDM (Figure 1A). Here, it is important to note that the comparison between human and mouse data is only informative, since the objective of this experiment was to confirm the gene expression of CD3Σ in the cell line.

Following, we evaluated the CD3 protein level by immunoblot analysis. We used a monoclonal antibody (mAbs) anti-CD3-Σ chain, which recognizes both human (h) and murine (m) CD3 proteins, and it was probed in the whole lysate obtained from RAW (m), CD14+ (h), MDM (h), CD4+ T cells (h), splenocytes (S, m) and bone marrow (BM, m) cells (Figure 1B). As expected, splenocytes and CD4+ T cells revealed a strong CD3 signal (Figure 1B, fourth and fifth lane). However, compared to splenocytes, CD14+ and MDM showed a weaker but prominent CD3 signal (Figure 1B, second and third lane, respectively) in contrast to the CD3-negative BM (Figure 1B, right lane). Thus, we confirmed that RAW cells express the CD3-ε chain by immunoblot analysis.

To date, we did not know if the CD3 could be found only on the cell surface, or if it could be accumulated intracellularly. Thus, RAW cells were prepared for flow cytometry using two mAbs that recognize both the CD3-Σ chain and are coupled to different fluorochromes. One was used for extracellular staining, and another was added after the cell permeabilization to identify the intracellular CD3. F4/80 and CD11b were included as myeloid cell markers (Figure 1C). As we expected, nearly all RAW cells were F4/80+ (98%). Extracellular staining demonstrated ~56% CD3+ RAW cells (min 54%, max 58%) (Figure 1D). With the co-staining, we confirmed that 56% of RAW cells were CD3+ when marked at the cell surface, ~20% were CD3+ when stained intracellularly (min 20%, max 29%), and 10% (min 9%, max 13%) were stained by both intracellular and extracellular CD3 antibodies (Figure 1E).

Previously, we reported that BCG infection increases the frequency of CD3+ myeloid cells [10,11]. For further validation of the CD3+ RAW cells, uninfected and BCG-infected (24 h, MOI:1) RAW cells were immunoprecipitated (IP) with one anti-CD3-Σ mAb and detected with a second anti-CD3-Σ mAb. In consonance with the previous technique, CD3-Σ chain was identified in the fraction of the IP-RAW cells but not in the supernatant (SN) fraction. The detection was independent of the infection status (Figure 1F). 

These results confirmed that the murine macrophage cell line RAW expresses CD3 at the transcriptional and protein levels. Moreover, CD3 expression was found both intracellularly and on the cell surface in uninfected and BCG-infected RAW cells.

### 3.2. The CD3 Complex Is Expressed Dynamically on the Cell Surface of RAW Cells

Previously, we reported that around 30% of RAW cells are CD3+ [10], and more than three decades ago, it was demonstrated that CD3–TCR complex expression is modulated dynamically on T cells (4). Therefore, we hypothesized that the difference in the frequency of CD3+ RAW cells between our previous and current study might be due to CD3 being expressed dynamically on RAW cells. 

**Figure 2:**
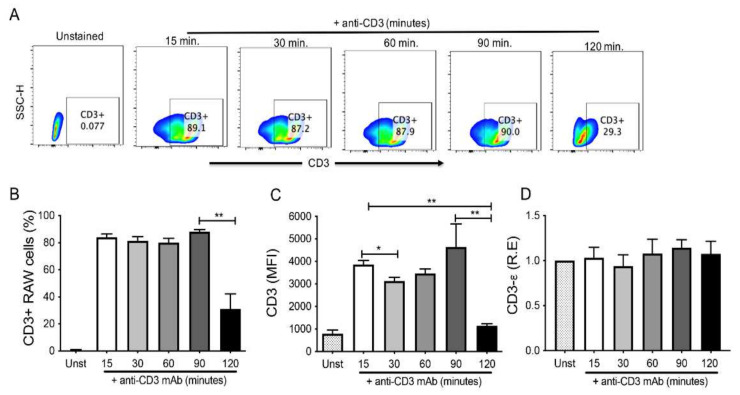
CD3 is endocytosed on RAW macrophages. (**A**) Representative dot plot showing analysis strategy to identify by flow cytometry the extracellular CD3 expression at different times after adding anti-CD3. (**B**) Frequency of CD3+ RAW at different indicated times. (**C**) Mean fluorescence intensity (MFI) of CD3 in RAW cells at indicated time points. (**D**) RNA was obtained from the RAW cells after anti-CD3 addition at different times to assess the relative expression (R.E) of CD3-Σ by qPCR. *n* = 5 independent experiments (for flow cytometry), *n* = 3 independent experiments performed by duplicate (for qPCR). Bars represent the mean value ± SD (to (**B**,**C**)) or the mean of means ± SEM (to (**D**)). Differences between times were analyzed using the Kruskal–Wallis and corrected for the Dunn test for flow cytometry data and one-way ANOVA followed by the Bonferroni test for qPCR. * *p <* 0.05, ** *p <* 0.01.

To confirm our hypothesis, RAW cells were probed with an anti-CD3-Σ chain mAb and analyzed by flow cytometry after 15, 30, 60, 90, and 120 min (Figure 2A). We found that the frequency of CD3+ RAW was constant for 90 min, followed by a significant drop to 27% after 120 min of incubation (Figure 2B).

Interestingly, in contrast to the mere cell count, we observed that the positive cells’ mean fluorescence intensity (MFI) was not constant throughout the experiment. The highest MFI was observed at 90 min and 15 min; meanwhile, at 120 min, the CD3 MFI decreased significantly (*p <* 0.05 versus 15 min; and *p <* 0.01 versus 90 min); similarly, at 30 min the MFI decreased compared to its level at 15 min (*p <* 0.01) (Figure 2C). At 60 min, the MFI tended to be lower compared to its level at 15 min; however, it was not statistically significant. The MFI clearly showed a dynamic cell surface expression of CD3 (Figure 2C).

To confirm that although RAW cells display changes in CD3 expression on the cell surface, they always maintain the capacity to express CD3, we evaluated the transcriptional level of CD3 in the same conditions. Our data show that at the transcriptional level, CD3 expression was not modified (Figure 2D). This result demonstrates that CD3 expression is modulated dynamically on the cell surface of RAW cells but remains constant at the transcriptional level.

### 3.3. CD3+ RAW Shows Higher Levels of NFAT-1 and pC-Jun Than CD3- RAW, and They Can Produce Proinflammatory Cytokines

**Figure 3:**
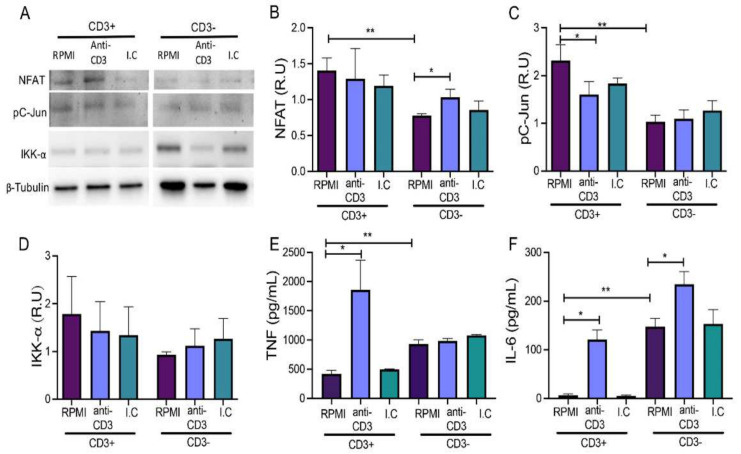
Anti-CD3 stimulation induces the production of TNF and IL-6 in CD3+ RAW cells. (**A**) Sorted RAW cells were used to evaluate NFAT, phosphorylated C-Jun (pC-Jun), and IKK-α by immunoblot. β-Tubulin was used as a loading control. CD3+ and CD3- RAW cells were cultured for 24 h under the following conditions: RPMI, anti-CD3, and isotype control (I.C). (**B**) Relative units (R.U) of NFAT-1, (**C**) pC-Jun, and (**D**) IKK-α in RAW CD3+ and CD3- cell lysates. TNF and IL-6 levels ((**E**,**F**), respectively) were measured in supernatants collected from CD3+ and CD3- RAW cells by ELISA. *n* = 3 independent experiments (for immunoblot) and *n* = 3 independent experiments performed in triplicate (for ELISA). Bars represent the mean value ± SD (**B**–**D**) or the mean of means ± SEM (**E**,**F**). Kruskal–Wallis and corrected for the Dunn test were used for immunoblot data and one-way ANOVA followed by Bonferroni test for ELISA. * *p <* 0.05, ** *p <* 0.01.

Diverse molecules are involved downstream of the CD3 activation, such as NFAT, c-Jun, which is associated with c-Fos to conform AP-1, and IKK-α, which is related to NF-κB activation [6,7,8,20]. Given that CD3 is dynamically expressed, it is conceivable that the fraction of CD3- RAW cells might at any other point-of-culture also be CD3+. To clarify this question, we evaluated if sorted CD3+ and CD3- RAW cells differ in pathways downstream of CD3. To this end, we treated RAW cells with an anti-CD3 mAb for 24 h and NFAT, phosphorylated c-Jun (pC-Jun), and IKK-α protein levels were evaluated by immunoblot (Figure 3A).

We found that NFAT was decreased at baseline near 50% in CD3- compared to CD3+ (*p <* 0.01), and interestingly, when CD3- were stimulated with anti-CD3 mAb, NFAT increased to near 20%, compared to RPMI (*p <* 0.05) (Figure 3B). Regarding the pC-Jun level, CD3+ expressed nearly twice as much as CD3- RAW cells at baseline (RPMI, *p <* 0.01), but when CD3+ RAW cells were stimulated with the anti-CD3 mAb, pC-Jun protein levels were reduced (*p <* 0.05) (Figure 3C). IKK-α level did not change in either of the two groups upon treatment, although at baseline, we observed slightly higher levels in CD3+ compared to the CD3- RAW cells; however, the differences did not reach statistical significance (Figure 3D).

To evaluate the significance of elevated NFAT levels in CD3- after the stimulus, we analyzed the levels of two proinflammatory cytokines in the supernatant that were produced efficiently by RAW cells when activated, namely TNF and interleukin (IL)-6. Remarkably, we observed that only CD3+ RAW and not CD3- RAW cells increased TNF production after stimulation with anti-CD3 mAb compared to basal condition (*p <* 0.05) (Figure 3E). However, at basal condition, CD3- RAW cells showed a higher TNF level compared to CD3+ RAW cells (*p <* 0.01) (Figure 3E). Regarding IL-6, both CD3+ and CD3- RAW cells produced a higher cytokine level after stimulation with anti-CD3 mAb than in the control conditions (Figure 3F). Additionally, we identified that CD3- cells also produced a higher level of IL-6 in control conditions than the basal level of CD3+ cells (*p <* 0.01). 

Together, these results suggest that although CD3- RAW cells express lower NFAT and pC-Jun levels than CD3+ RAW cells at baseline, they produce higher TNF and IL-6, but CD3+ RAW cells produce TNF and IL-6 efficiently as a response to the anti-CD3 stimuli.

### 3.4. 24 h Is a Long Time to Observe Downstream Molecules in Total RAW Cells

**Figure 4:**
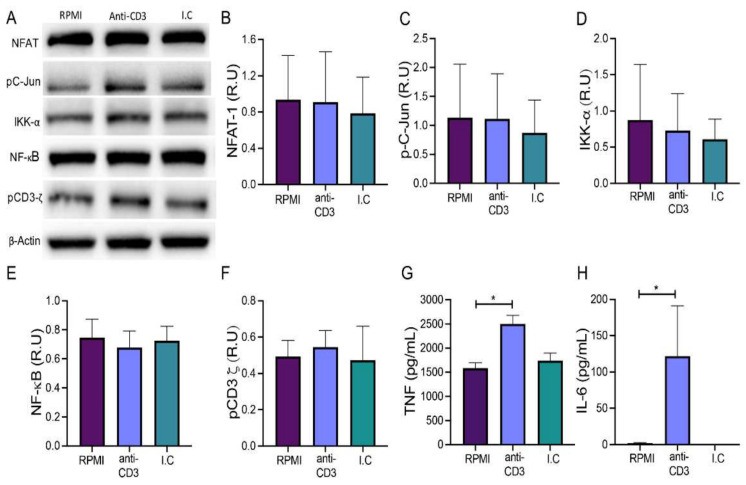
The anti-CD3 monoclonal antibody does not induce activation in total RAW cells. (**A**) Immunoblot evaluation of proteins involved in the CD3 signaling pathway; NFAT, phosphorylated C-Jun (pC-Jun), IKK-α, NF-κB and phosphorylated CD3-ζ chain (pCD3-ζ), β-Tubulin as loading control in lysates of RAW cells cultured for 24 h under the following conditions: RPMI (control), anti-CD3 (stimulus), and isotype control. Relative units (R.U) of NFAT, pC-Jun, IKK-α, NF-κB, and pCD3-ζ in RAW cell lysates ((**B**–**F**), respectively). Levels of TNF and IL-6 were measured in supernatants collected from total RAW cells by ELISA sandwich assay ((**G**,**H**), respectively). *n* = 5 independent experiments (immunoblot) and *n* = 5 independent experiments performed in triplicate (ELISA). Bars represent the mean value ± SD (**B**–**F**) or the mean of means ± SEM (**G**,**H**). Kruskal–Wallis and corrected for the Dunn test were used for immunoblot data and one-way ANOVA followed by Bonferroni test for ELISA. * *p <* 0.05.

Since cell surface CD3 expression is not constant in RAW cells, unsorted RAW cells were cultured with anti-CD3 mAb and an isotype control to evaluate if under this condition they can also be activated by anti-CD3 stimulus. In addition, NFAT, pC-Jun, and IKK-α were assessed by Western blot, and the evaluation of NF-kB and the phosphorylated CD3-ζ chain (pCD3-ζ) were included (Figure 4A). 

Our data showed that the level of NFAT, pC-Jun, IKK-α, NF-κB, and pCD3-ζ were not modified after 24 h of anti-CD3 stimulus (Figure 4B–F, respectively). To confirm that the cells were activated at all, we determined TNF and IL-6 protein levels in the supernatants of the treated cells. We found increased TNF and IL-6 levels after stimulation with anti-CD3 mAb compared to vehicle control (2501, min 1900-max 2932 versus 1586, min 1346-max 1976 ng/ml, respectively, *p <* 0.05; 122, min 0-max 375 versus 2, min 0-max 4 ng/ml, respectively, *p <* 0.05) (Figure 4G,H). Together, these results suggest that total RAW cells with anti-CD3 stimuli produce proinflammatory cytokines; however, downstream molecules need to be investigated at earlier time points.

### 3.5. BCG In Vitro Infection Maintains the CD3 Expression and Induces a Proinflammatory Profile in RAW Cells

Previous reports suggested that CD3+ myeloid cells play an essential role during mycobacterial infection, and tmTNF also is necessary to maintain this cell subpopulation. Therefore, to evaluate if BCG infection affects the expression of CD3 and tmTNF, we followed a previous experimental design where the BCG infection induces the delivery of TNF by RAW cells [21]. 

**Figure 5:**
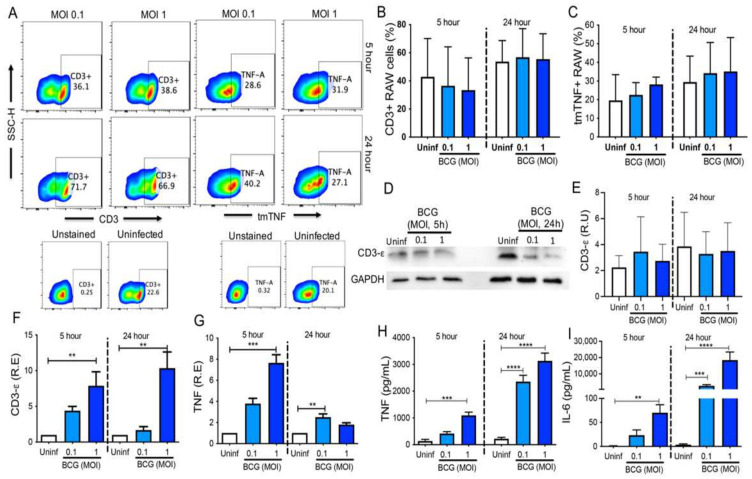
RAW cells increase CD3 and TNF at the transcriptional level after in vitro BCG infection. (**A**) Representative dot plot showing analysis strategy to identify, by flow cytometry, the CD3 and transmembrane TNF (tmTNF) expression in RAW cells uninfected (Uninf) and infected in vitro with BCG MOI 0.1 and 1 during 5 and 24 h. Controls of unstained and uninfected cells are shown down to the respective molecule. Percentage of CD3+ and tmTNF+ RAW cells evaluated by flow cytometry ((**B**,**C**), respectively). (**D**) Immunoblot evaluation of CD3-Σ in lysates of uninfected and infected RAW cells. GAPDH was used as a loading control. (**E**) Relative units (R.U) of CD3-ε and TNF expression evaluated by immunoblot, and relative expression (R.E) of CD3-ε and TNF genes assessed by qPCR in uninfected and infected RAW cells ((**F**,**G**), respectively). Quantification of soluble TNF and IL-6 by ELISA in culture supernatants from uninfected and infected RAW cells ((**H**,**I**), respectively). *n* = 7 independent experiments (for flow cytometry), *n* = 5 independent experiments (for immunoblot), and *n* = 7 independent experiments performed in triplicate (for qPCR and ELISA). Bars represent the mean value ± SD (**B**,**C**,**E**) or the mean of means ± SEM (**F**–**I**). Kruskal–Wallis and corrected for the Dunn test were used for flow cytometry and immunoblot analysis, and one-way ANOVA followed by Bonferroni test for qPCR and ELISA data. ** *p <* 0.01, *** *p <* 0.001, **** *p <* 0.0001.

First, we evaluated the RAW CD3 and tmTNF cell-surface expression by flow cytometry at 5 and 24 h post-infection with BCG (MOI 0.1 and 1) (Figure 5A). The frequencies of CD3+ and tmTNF+ RAW cells (Figure 5B,C; respectively) were not modified by the BCG infection. To confirm these findings, we quantified the CD3-Σ protein level in RAW cells by immunoblot analysis (Figure 5D). We could confirm that the infection neither modified the frequency of CD3+ cells nor the total CD3 protein levels. We could, however, again observe the different CD3 protein expression in uninfected cells at 5 and 24 h after infection, supporting our hypothesis of dynamic CD3 expression (Figure 5E).

Moreover, CD3-Σ chain and TNF were evaluated at the transcriptional level, and our results showed that the CD3-Σ chain increased significantly with BCG MOI 1, and it was observed at both 5 and 24 h post-infection (*p <* 0.01) (Figure 5F). TNF was increased with the MOI 1 at 5 h (*p <* 0.001) and normalized at 24 h, whereas BCG MOI 0.1 did not increase at 5 h, but at 24 h, it started to increase (*p <* 0.01) (Figure 5G).

Finally, we evaluated the levels of secreted TNF and IL-6 in the supernatant. As expected, MOI 1 increased the TNF secretion at both 5 h (*p <* 0.01) and 24 h (*p <* 0.0001), and MOI 0.1 increased soluble TNF levels at 24 h only (*p <* 0.0001) (Figure 5H). Similarly, the IL-6 level was increased by MOI 1 at both 5 and 24 h (*p <* 0.001 and *p <* 0.0001, respectively), whereas MOI 0.1 increased it only at 24 h (*p <* 0.0001) (Figure 5I).

Together, these results suggest that BCG infection increases the CD3-Σ chain mRNA level, although it could not be observed at the protein level; the increase in TNF level was confirmed at both the protein and transcriptional levels.

## 4. Discussion

CD3+ myeloid cells are a small subpopulation, which can be found in peripheral blood (CD3+ monocytes) and tissue (CD3+ macrophages); however, under nonpathological conditions, only 12–15% of monocytes can be differentiated into CD3+ macrophages [3,12,16]. Reports indicated that these cells can be activated by a CD3-dependent pathway to deliver proinflammatory cytokines by an—up till now—unknown pathway.

Although the primary cell culture is used as a model to study the cell signaling because it can provide information closer to what happens in an in vivo system, the low frequency of CD3+ myeloid cells is a significant limitation to design deep studies that allow us to clarify the CD3-dependent signaling. Thus, using a myeloid cell line to identify cell signaling is an alternative to screening molecules involved in the CD3-dependent cell activation; this information could be posteriorly translated to primary cultures of human cells to identify key molecules. In this study, we demonstrated the use of RAW cells as a new approach to studying the CD3 signaling in myeloid cells. Here, it is essential to note that a human monocyte cell line was also used; however, we could not identify the CD3 expression (data not shown).

Reports have described that CD3+ macrophages secrete proinflammatory cytokines such as TNF, IFN-γ, IP-10, CCL-2, and IL-1β, among others, by a CD3-dependent pathway [11]. However, it is still unclear how the signal after CD3 stimulus is transduced. This knowledge is imperative to identify mechanisms to regulate its function under the different pathologies where this cell subpopulation has been identified. Probably, it also can provide highlights regarding its origin.

The role of macrophages is not limited to pathological conditions; they are necessary to repair tissue even under sterile conditions. We demonstrated that naïve mice have CD3+ myeloid cells in the lung tissue [11]; in this regard, new questions are opened. For instance, it is necessary to know if a proinflammatory stimulus is required to induce CD3+ macrophage proliferation in situ, or if the monocytes’ recruitment is needed to increase the frequency of these cells during an inflammatory process.

**Figure 6:**
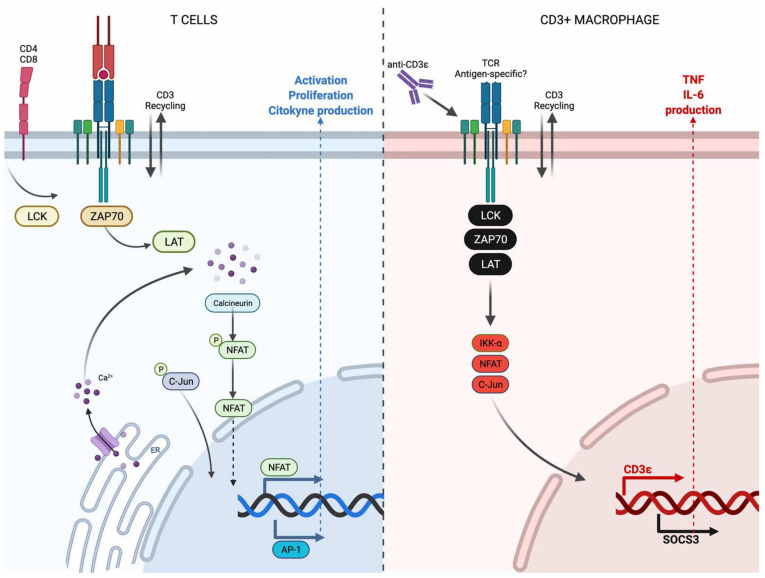
Current knowledge of the CD3 downstream signaling in T cells and macrophages. T cells express the CD3/TCR complex, and these cells are activated after the TCR recognizes a specific antigen. Lck, ZAP70 and LAT are recruited to stimulate the nuclear factors NFAT and AP-1, to finally favor functions such as activation, proliferation, and cytokine production (**left**). CD3+ macrophages also express CD3, and some of them are TCR+. At present, it is still unclear if this TCR is antigen-specific, but these cells can be activated by anti-CD3 stimulus. Lck, ZAP70, LAT, IKK-a, NFAT and C-Jun play a role in activating the CD3+ macrophages and favor the delivery of pro-inflammatory cytokines such as TNF and IL-6 (**right**). The figure was created in BioRender.

CD3 expression has been almost exclusively associated with lymphoid cells, and the CD3-dependent signaling to favor T cell activation is well understood. Following the recent evidence reviewed by Ashouri JF [22], it is summarized in Figure 6 (left). Upon recognition by the TCR, intracellular downstream signals are initiated, and Lck kinase induces the phosphorylation of the CD3-ζ chain to favor the ZAP70 recruitment. Subsequently, it interacts with LAT to actively recruit LAT to the activated ZAP70. To regulate the effector functions of T cells, such as the delivery of cytokines, other molecules play an essential role; for example, the activated T cell increases its calcium level, increasing the function of calcineurin to dephosphorylate NFAT; then, it is translocated into the nucleus to regulate the expression of cytokines [23]. Similarly, studies have shown that the c-Jun/c-Fos heterodimer functions downstream of the TCR to induce the synthesis of cytokines [24].

In contrast to T cells, information on the CD3-signaling pathways in myeloid cells is limited. This work identified that RAW murine macrophages express CD3 at transcriptional and protein levels. Like lymphocytes, the CD3 expression on the cell surface is cycling even though it remains stable at the transcriptional level. Thus, our main aim was to support the use of this cell line as an option to design experimental strategies that allow us to identify the CD3-signaling pathway in myeloid cells. Although it is possible that the CD3-signaling in myeloid cells is not the same between humans and mice, this study can provide valuable information to facilitate future studies in human cells. 

In this model, the anti-CD3 stimulus induces the delivery of the proinflammatory cytokines TNF and IL-6, but it is important to note that two different profiles could be observed, and that they were dependent on whether the RAW cells were mixed (CD3+/CD3−) or enriched. At baseline purified CD3+ showed a higher level of NFAT and pC-jun than did CD3-cells, but simultaneously, they delivered lower TNF and IL-6 levels, suggesting that the CD3 expression provides a different profile of activation. On the other hand, when all RAW cells were together, we did not identify changes in the involved molecules in the CD3-dependent pathway; we speculate that this was because the CD3 cell surface expression in RAW cells was not constant. So, the specific moment when the cell is activated by a CD3-dependent pathway is variable; consequently, these changes can be masked by the effect of CD3. Moreover, we cannot exclude the possibility that 24 h was too late to see differences in protein levels downstream of CD3 activation when heterogeneous RAW cultures were used because although we did not observe differences in the downstream molecules, we confirmed that at 24 h with the anti-CD3 stimulus, TNF and IL-6 were still very abundant in the medium supernatant.

In Figure 6 (right), we summarized the current evidence for CD3 signaling in myeloid cells; the names of the new molecules identified in this work are indicated in red, and the names of molecules provided by previous reports are indicated in black. Thus, at present, it is still unclear if the TCRs expressed by some CD3+ myeloid cells are antigen-specific and if they can initiate phosphorylation of the CD3 chains, but we identified that this CD3 expresses both Σ and ζ chains. Moreover, it has been described that human macrophages constitutively express Lck, ZAP70, and LAT. Due to anti-CD3 stimulus, these cells produce proinflammatory cytokines, probably by a SOCS3-dependent pathway [3,11]. Our current results also showed that the CD3+ RAW cells display a high level of NFAT, pC-Jun, and IKK-a, suggesting that they could be participating in the CD3 downstream signaling, at least in mouse cells; future studies with human cells are necessary.

Previous reports indicated that in response to BCG infection, the frequency of CD3+ myeloid cells is increased in vivo. This was observed for cells of both human and murine origin [3,11,12]. In this study, we did not identify an increased frequency of CD3+ RAW cells after BCG infection, but we observed that CD3 is increased at the transcriptional level 5 h post-infection (MOI 1). Therefore, the discrepancy in CD3 expression on the cell surface could be due to these cells being activated (because of the same infection). Consequently, the CD3 recycling is constantly happening, and it is a limitation to observation of the frequency of CD3+ RAW cells under this condition.

This study provides evidence that RAW cells are suitable as a model to study the CD3 signaling in myeloid cells. Identifying molecules involved in this pathway is necessary to modulate the function of the CD3+ myeloid cells observed in diverse pathologies.

This study is not free of limitations; perhaps the most important is that we used a cell line with a murine origin. However, we provided a suitable alternative to study CD3+ myeloid cell activation signaling in more detail. This model can be helpful to clarify questions such as the optimal timing for activation of CD3+ macrophages if there are co-stimulation mechanisms homologous to CD28 in macrophages, how the signal is amplified, and if these macrophages display a type of hybrid signaling in which innate receptors participate, among others. Finding the answer to these questions will highlight if there are pre-requisites for the complete activation of human CD3+ macrophages and which molecules may be involved in this pathway.

## Data Availability

The authors confirm that the raw data supporting the conclusions of this study are included in the manuscript. The corresponding author will provide more information, upon reasonable request, to any qualified researcher.

## Appendix A

**Table A1:** Information on the antibodies used in this study.

Antibody	Company	Clone	Ref. Number
anti phospho CD3 zeta	NOVUSBIO	EM-54	NBP2-37708
anti mouse CD3 epsilon	eBioscience	145-2C11	14-0031-850
PE anti-mouse CD3 epsilon	BioLegend	145-2C11	100308
PE Armenian Hamster IgG isotype control	Biolegend	HTK888	400907
PE/Cy7 anti-mouse CD3 epsilon	BioLegend	145-2C11	100320
PE Armenian Hamster IgG isotype control	Biolegend	HTK888	400922
Pe/Cy5 anti-mouse F4/80	BioLegend	BM8	123112
FITC anti-mouse/human CD11b	BioLegend	M1/70	101206
Pe/Cy5 anti-mouse TNF-a	BioLegend	MP6-XT22	506323
TruStain FcX™ (anti-mouse CD16/32) Antibody	BioLegend	93	101320
Ultra-LEAF(TM) Purified anti-mouse CD3 antibody	BioLegend	17A2	100239
Ultra-LEAF Purified Rat IgG2b kappa Isotype Ctrl rat	BioLegend	RTK4530	400643
anti NF kappa B p65	Santa Cruz Biotech.	Polyclonal	sc-372
anti IKK-a	Santa Cruz Biotech.	C-6	sc-166231
anti alpha tubulin	Genetex	Polyclonal	GTX112141
anti beta actin	Genetex	Polyclonal	GTX109639
anti GAPDH	Genetex	Polyclonal	GTX100118
anti Phospho-c-Jun (Ser73)	Cell Signaling	D47G9	3270s
anti NFAT1	Cell Signaling	Polyclonal	4389s
anti mouse TNF-α Rabbit Antibody	Cell Signaling	D2D4	11948T
anti-CD3e Molecule, epsilon	Antibodies-Online	Polyclonal	ABIN343654
Goat Anti-Mouse IgG Polyclonal antibody HRP Conjugated	R&D Systems	Polyclonal	HAF007
Goat Anti-Rabbit IgG HRP Conjugated	R&D Systems	Polyclonal	HAF008
